# Response to correspondence received on “New perspectives on endocrine therapy suitability for hormone receptor-positive metastatic breast cancer in clinical practice”

**DOI:** 10.1016/j.breast.2026.104866

**Published:** 2026-07-23

**Authors:** Hope S. Rugo

**Affiliations:** City of Hope Comprehensive Cancer Center, 1500 Duarte Rd, Duarte, CA, 91010, USA

Dear Dr Yuen,

Many thanks your correspondence regarding our manuscript *“New perspectives on endocrine therapy suitability for hormone receptor-positive metastatic breast cancer in clinical practice”*, currently published in *The Breast*. We appreciate your thoughts and the time taken to raise this correspondence.

To address your first point, we agree and do not consider 1–10% ER staining to be a subset of ER-negative. The cited ASCO/CAP guidance states: “… it is reasonable for oncologists to discuss the pros and cons of endocrine therapy with patients whose cancers contain low levels of ER by immunohistochemistry (IHC; 1–10%)”. In line with this guidance, we suggest in our paper that clinicians should carefully consider the suitability of patients with low levels of staining for ER, defined as 1–10%.

To the second point, our intention was to highlight that Allred score is one of several methods that may be cited when identifying estrogen receptor status. At the start of our paper, we give an overview of different markers associated with response to endocrine-based therapy, and do not suggest that any of these measures should be used in isolation. Rather, one of the key discussion points is that it is necessary to use multiple markers in conjunction in order to make well-informed treatment decisions. However, we agree that the sentence should be updated as follows: “For ER positive tumors (≥1% tumor nuclei positive) an Allred score >2 is interpreted as estrogen receptor-positive and may be reported complementarily to the percentage of cells staining and intensity” and have requested this corrigendum.

Yours sincerely, Prof. Hope S Rugo

